# Downregulation of Enhancer of Zeste Homolog 2 (EZH2) is essential for the Induction of Autophagy and Apoptosis in Colorectal Cancer Cells

**DOI:** 10.3390/genes7100083

**Published:** 2016-10-03

**Authors:** Yizhou Yao, Hao Hu, Yong Yang, Guoqiang Zhou, Zengfu Shang, Xiaodong Yang, Kang Sun, Shenghua Zhan, Zhengyuan Yu, Peiyao Li, Guofeng Pan, Liang Sun, Xinguo Zhu, Songbing He

**Affiliations:** 1 Department of General Surgery, The First Affiliated Hospital of Soochow University, Suzhou 215006, China; yaoyizhou2014@163.com (Y.Y.); lidechun1955@163.com (H.H.); magicy@126.com (Y.Y.); 1030705022@suda.edu.cn (G.P.); 1130705026@suda.edu.cn (L.S.); zxg45@hotmail.com (X.Z.); 2 Department of Gastrointestinal Surgery, Changshu No.2 Hospital, Suzhou 215500, China; chowgq@sina.com; 3 Department of Radiation Medicine, Medical College of Soochow University, Suzhou 215006, China; zengfu.shang@suda.edu.cn; 4 Department of General Surgery, The Second Affiliated Hospital of Soochow University, Suzhou 215006, China; wjyxd@163.com; 5 Department of General Surgery, The Affiliated Hospital of Jiangsu University, Zhenjiang 212001, China; doctorsunkang@126.com; 6 Department of Pathology, The First Affiliated Hospital of Soochow University, Suzhou 215006, China; hesongbing1979@suda.edu.cn; 7 Department of Oncology, The First Affiliated Hospital of Soochow University, Suzhou 215006, China; strongeryy1985@163.com; 8 Medical Engineering and Maintenance Center, Chinese PLA General Hospital, Beijing 100853, China; li_peiyao@163.com

**Keywords:** EZH2, shRNA, DZNep, autophagy, apoptosis, colorectal cancer

## Abstract

Increasing evidence indicates that elevated expression of enhancer of zeste homolog 2 gene (EZH2) in many human malignant tumors acts a significant role in the oncogenic process. However, the underlying molecular mechanism is still unclarified. It is evident that apoptosis and autophagy of tumor cells is crucial for the tumorigenesis and progression of cancer, however, the exact role of EZH2 plays in apoptosis and autophagy has not been fully elucidated in colorectal cancer (CRC). Our previous study found that the expression level of EZH2 was higher in CRC tumor tissues than in the paired normal tissues using immunohistochemical analysis. We also recently found that the autophagy-related gene-related protein Ambra1 plays an important role in the autophagy pathway in CRC cells. In this study, mRNA and protein expression of EZH2 in four CRC cell lines were tested at first and RKO and HCT116 cells showed the highest levels among them. Here we transfected with EZH2-shRNA, or added DZNep (an EZH2 inhibitor) to RKO and HCT116 cells in order to detect the effect of EZH2 on autophagy via determining the change of the protein expression of LC3 and Ambra1. The outcome indicated an obvious decrease of autophagy level in cells transfected with EZH2-shRNA or DZNep. We also found the apoptotic rate of cells was elevated significantly after downregulation of EZH2. In addition, compared to control group, CRC cells transfected with EZH2-shRNA or added DZNep revealed a significantly increased G1 cell cycle rate and an obvious decrease in the G2 cell cycle rate. Further analysis showed that knockdown of EZH2 induced cell-cycle arrest in CRC cells. Meanwhile, downregulation of EZH2 in CRC cells induces autophagy and apoptosis. Taken together, our results suggest that EZH2 plays a critical role in autophagy and apoptosis in the progression of CRC, which potentially facilitates the development of an ideal strategy for combating colorectal cancer.

## 1. Introduction

Colorectal cancer (CRC) is one of the most common cancers worldwide [1], which represents the second leading cause of cancer-related death in the Western countries [2]. Although great advancements in early diagnosis and treatment of CRC have been made, incidence and mortality remain high [3,4]. Therefore, it is very urgent to find an effective therapy to fight this lethal disease. It is reported that several signal pathways contribute to and influence the initiation and progression of CRC, and molecular target agents including epigenetic target agents may be an underlying novel therapeutic strategy for acquiring profiles on survival of CRC patients.

Enhancer of zeste homologue 2 gene (EZH2), a core component of the human polycomb repressive complex 2 (PRC2), is deemed to tri-methylate histone H3 at lysine 27 and elicit gene silencing [5]. Increasing evidence indicates that EZH2 is associated with a number of cancers, including nasopharyngeal esophageal cancer, breast cancer, lung cancer and bladder cancer [6,7,8,9]. Moreover, overexpression of EZH2 indicates the tumor more aggressive and is related to poor prognosis [10,11,12]. In our previous immunohistochemical research, we found that expression of the EZH2 was significantly higher in tumor tissues than the paired normal tissues, and EZH2 might facilitate tumorigenesis in CRC cells [13]. Therefore, EZH2 represents a promising potential target for combating CRC. It has been recognized that apoptosis and autophagy of tumor cells is crucial in tumorigenesis and progression of cancer, however, the exact role EZH2 plays in apoptosis and autophagy has not been fully elucidated in CRC.

In this study, we further investigated the effect of downregulation of EZH2 on CRC cells. We transfected EZH2-short hairpin RNA (shRNA) into CRC cell lines and used an EZH2 inhibitor 3-Deazaneplanocin A (DZNep), to examine the potential involvement of autophagy and apoptosis. We further explored influence on cell cycle after downregulation of EZH2. Our outcome in this study indicates that blocking expression of EZH2 would be an ideal strategy for combating colorectal cancer. Thus, more research should be done to get a better understanding of epigenetic mechanisms and the influence of EZH2 targeted agents on potential survival benefit in CRC patients. This may promote a novel clinical strategy for CRC treatment.

## 2. Materials and Methods

### 2.1. Cell Lines and DZNep

The human CRC cell lines (RKO, HCT116, SW480 and CCL224) were purchased from the Chinese Academy of Sciences (Shanghai, China) and cultured in the six-well dishes at the concentration of 1.5 × 10^5^/well as per the instructions of ATCC. All four cell lines were maintained in RPMI-1640 (Invitrogen, Carlsbad, CA, USA) supplemented with 10% fetal bovine serum (FBS; Sijiqing Biological Engineering Materials Co., Hangzhou, China) at 37 °C with 5% CO_2_. DZNep, an EZH2 inhibitor, was purchased from sigma-Aldrich (SML0305, Sigma, St. Louis, MO, USA), and it was dissolved in water. The solution is stored in −20 °C with the concentration of 5 mg/mL. The RKO and HCT116 cells were treated with DZNep 24 h after culturing. After another 72 h, the total protein and RNA were extracted.

### 2.2. Construction of shRNA and Cell Transfection

shRNA sequences were synthesized by GENE CHEM (Shanghai, China). According to the EZH2 mRNA sequence in GenBank, three 19bp targeting sequences were designed. The relevant shRNA template oligonucleotide sequences are shown in Table 1. They were all confirmed by DNA sequencing. After plasmid transfection, LV‑EZH2-shRNA was harvested. The lentivirus was titrated to 1 × 10^9^ TU/mL. RKO and HCT116 cells were cultured at the concentration of 5 × 10^4^/well in a six-well dish for 24 h, then we added lentivirus and polybrene to the culture medium according to multiplicity of infection (MOI). We changed the medium 16 h after transfection, and the subsequent experiments were performed 72 h later.

### 2.3. Quantitative Real-Time Reverse Transcription PCR (QRT-PCR)

We performed QRT-PCR to further investigate the mRNA expression of EZH2 in RKO and HCT116 cells. Total RNA was extracted using TRIzol (Invitrogen, Life Technologies, Carlsbad, CA, USA) and 5 μg of RNA was used to synthesize cDNA according to the manufacturer′s instructions. The PCR conditions consisted of 5 min at 95 °C 1 cycle，30 s at 95 °C，30 s at 55 °C，30 s at 72 °C and 7 min at 72 °C 40 cycles. The sequences for sense (S) and antisense (AS) primers are as follows: EZH2: GTACACGGGGATAGAGAATGTGG (S), GGTGGGCGGCTTTCTTTATCA (AS); GAPDH: TGACTTCAACAGCGACACCCA (S), CACCCTGTTGCTGTAGCCAAA (AS). Every experiment was performed three times and the samples were tested in triplicate. The relative fold changes in mRNA expression were calculated using the 2^–ΔΔCT^ method, where the average of ΔCT values for the amplicon of interest was normalized to that of GAPDH, and compared with the control specimens.

### 2.4. Western Blot

The whole proteins were extracted from RKO and HCT116 cells, and then they were lysed with RIPA (Beyotime Inc., NanTong, China) according to the manufacture′s protocol. The supernatants were collected after centrifugation at 16,000 r/min for 15 min at 4 °C. 40 μg of the protein was separated from each sample preparation using the SDS-PAGE and then transferred onto polyvinylidene difluoride (PVDF) membranes. Afterwards, the membranes were blocked and incubated respectively with rabbit anti-human EZH2 antibody (1:1000, CST, Boston, MA, USA), rabbit anti-human LC3A antibody (1:1000, CST), rabbit anti-human Ambra1 antibody (1:1000, Abcam) at 4 °C overnight. The membranes were subsequently washed three times with the TBST for 10 min and incubated with secondary anti-body (1:1000, Santa Cruz, CA, USA) at 37 °C for 1 h. Following this, the membranes were washed three times with Tris-buffered saline with Tween (TBST). Bands were finally visualized by using an ECL + Plus^TM^ Western blot system kit (Amersham, GE Healthcare, Chicago, IL, USA). All Western blots shown were representative results obtained from at least three independent experiments.

### 2.5. Cell Proliferation Assay

At a logarithmic phase, RKO and HCT116 cells were divided into three groups (untreated, control-shRNA, EZH2-shRNA). In the medical experiment, the CRC cells were divided into two groups (untreated, DZNep). At 6 h after transfection, RKO and HCT116 cells were digested, re-suspended, and inoculated into 96-well dishes. After 24, 48, and 72 h of incubation, cells were stained with 20 μL 3-(4, 5-Dimethylthiazol-2-yl)-2,5-diphenyltetrazolium bromide Methylthiazolyl tetrazolium (MTT) solution (5 mg/mL) at 37 °C for 4 h. Then supernatants were removed and formazan crystals were dissolved in 150 μL dimethyl sulfoxide (DMSO). The reaction product was quantified by measuring the optical density using test wavelength for 490 nm at room temperature. Every experiment was performed three times and the samples were tested in triplicate.

### 2.6. Cell Migration Assay

Cell migration assays were performed using a transwell permeable supports system (Corning, New York, NY, USA). RKO and HCT116 cells that were under different treatments were incubated in medium. After 24 h of transfection, 2 × 10^4^/well RKO and HCT116 cells were resuspended in serum-free DMEM medium and seeded into the Transwell inserts uncoated with growth factor-reduced Matrigel (BD Biosciences, Bedford, MA, USA), whereas each lower chamber was filled with 500 μL complete culture mediun. After 24 h of incubation at 37 °C, the cells on the upper side of the insert filter were completely removed by wiping with a cotton swab, and the cells that had invaded were fixed in methanol and stained with 0.1% crystal violet. The cells were counted manually under an inverted microscope on five random fields (scale bar = 200 μm). Every experiment was performed three times.

### 2.7. Cell Apoptosis Analysis

Cell apoptosis assays were detected by Annexin V-PI staining. Cells receiving different treatments were trypsinized with 0.25% trypsin in the absence of EDTA. The collection was washed with sufficient PBS twice and resuspended in 500 μL of binding buffer at a concentration of 0.2–1.0 × 10^6^ cells/mL. The samples were then double stained with 5 μL Annexin V and 5 μL PI at room temperature for 15 min in darkness. The samples were subsequently analyzed using a flow cytometer. Every experiment was performed three times.

### 2.8. Cell Cycle Analysis

The RKO and HCT116 cells that were transfected with control-shRNA or EZH2-shRNA or added DZNep were harvested after 5 days, when covered about 80%. The cells were digested with trypsin enzyme, and then the suspension was washed with D-Hanks and fixed with 75% ethanol on ice for 2 h. The cells were centrifuged at 1300 r/min for 5 min, and then washed with D-Hanks twice. PI (50 μg/mL) and 500 μL of Rnase (100 μg/mL) were added, and then cells were incubated in darkness at 4 °C for 15 min followed by analysis on a fluorescence activated cell sorting (FACS) can apparatus. Every experiment was performed three times.

### 2.9. Statistical Analysis

The SPSS17.0 software (SPSS Inc., Chicago, IL, USA) was used for data analysis. The data were presented as the mean ± standard deviation (SD). Significant differences between groups were compared using the *t*-test. *p* < 0.05 was considered statistically significant.

## 3. Results

### 3.1. EZH2 Is Over-Expressed in CRC Cells

**Figure 1:**
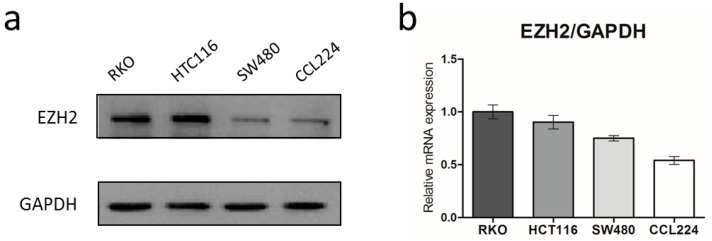
Expression of enhancer of zeste homolog 2 (EZH2) in colorectal cancer (CRC) cell lines. EZH2 protein and mRNA expression (**a,b**) in four CRC cell lines (RKO, HCT116, SW480, CCL224) detected by Western blot and QRT-PCR. The bar graphs represent the GAPDH-normalized EZH2 mRNA levels. Error bars represent SD (n = 3).

We recently reported that EZH2 is highly expressed in human CRC tissues by Immunohistochemical (IHC) staining. However, EZH2 was absent or weakly expressed in the paired non-tumor tissues. In order to confirm the protein and mRNA expression of EZH2 in CRC cell lines (RKO, HCT116, SW480, and CCL224), we first performed Western blot and QRT-PCR in the present study. The results showed that all the four cell lines expressed relatively high level of EZH2 and RKO and HCT116 cells showed the relative elevation among them, which were used for further studies (Figure 1).

### 3.2. EZH2-shRNA and DZNep Inhibited the Expression of EZH2 mRNA and Protein in RKO and HCT116 Cells

**Figure 2:**
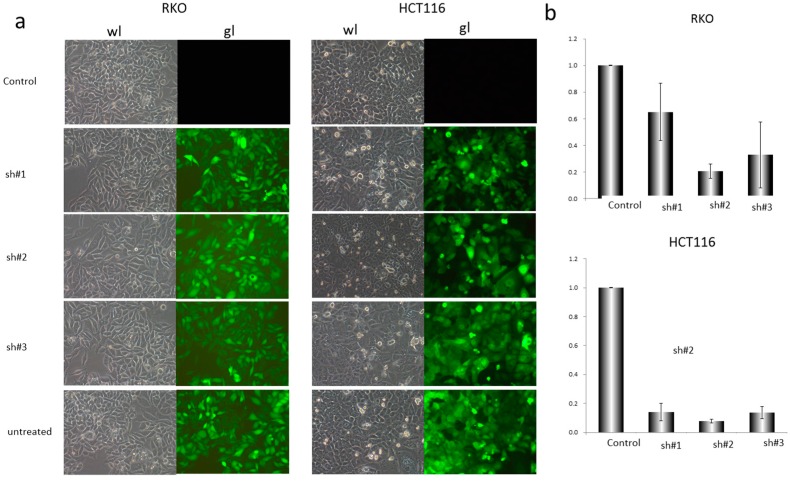
Deletion of EZH2 gene downregulated the expression of EZH2 in RKO and HCT116 cells by shRNA. (**a**) The white and fluorescence microscope pictures of RKO and HCT116 cells transfected respectively with 0.5 μL control-shRNA and EZH2-shRNA of three different sequences, represented as sh#1, sh#2, sh#3 (200×); (**b**) Relative expression of EZH2 mRNA in four groups of RKO and HCT116 cells (control-shRNA, sh#1, sh#2, sh#3) detected by QRT-PCR. The data were normalized to the level of GAPDH mRNA. Error bars represent SD (n = 3).

**Figure 3:**
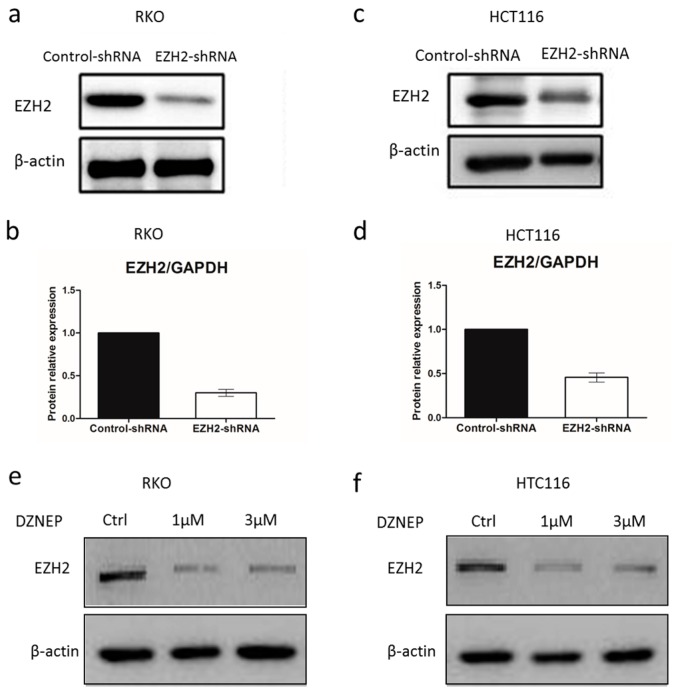
The knockdown efficiency of EZH2. The protein and mRNA levels of EZH2 expression in control-shRNA and EZH2-shRNA in RKO and HCT116 cells were tested by western blot (**a**,**c**) and QRT-PCR (**b**,**d**). Error bars represent SD (n = 3). HCT116 cells were treated with DZNep (1 or 3 μmol/L) for 72 h. EZH2 protein expression was analyzed by Western blot in RKO (**e**) and HCT116 (**f**) cells.

Next, we examined the infection efficiency of EZH2-shRNA lentiviral vector plasmid on CRC cells by treating cells with 0.1 μL, 1 μL or 10 μL lentivirus for transfection. After 72 h, the fluorescence intensities were observed under fluorescence microscope (Figure 2a), and then 0.5 μL was selected for further study. To test if EZH2 can be targeted by siRNA, three shRNA constructs (sh#1, sh#2 and sh#3) were designed to act on different regions of the EZH2 mRNA sequence (Table 1). RKO and HCT116 cells were transfected with control-shRNA or EZH2-shRNA. As presented in Figure 2a,b, sh#2 showed highest deletion among them. Afterwards, we explored the impact of shRNAs on silencing EZH2 expression in RKO and HCT116 cells. At 72 h after transfection with sh#2, the total protein and mRNA of all transfected and un-transfected cells were extracted and analyzed by Western blot and QRT-PCR. As shown in Figure 3a–d, EZH2 expression was distinctly decreased at mRNA and protein levels in transfected cells with EZH2-shRNA compared with control-shRNA. Thus, the results showed that the specific shRNA to EZH2 effectively suppressed the expression of EZH2 in RKO and HCT116 cells. In addition, DZNep, an EZH2 inhibitor, was performed in CRC cell lines for 72 h at the concentration of 1 or 3 μmol/L. Moreover, the protein expression of EZH2 in RKO and HCT116 clearly decreased.

### 3.3. Effects of EZH2 Downregulation on the Growth and Migration of RKO and HCT116 Cells

**Figure 4:**
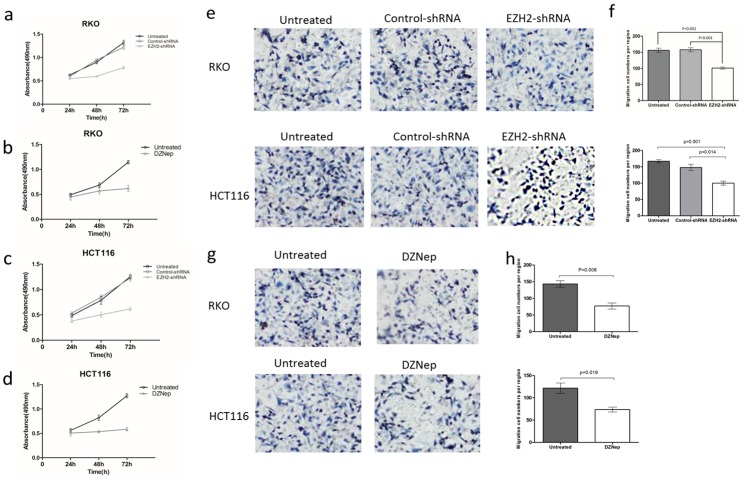
EZH2-shRNA inhibited proliferation and migration of RKO and HCT116 cells. MTT assay of RKO (**a**,**b**) and HCT116 (**c**,**d**) cells with EZH2 knockdown or 3 μmol/L DZNep. Curves of RKO and HCT116 cells growth after 24, 48, 72 h by different treatments. Error bars represent SD (n = 3). Cell migration assay of RKO and HCT116 cells with downregulation of EZH2 (**e**,**f**) or 3 μmol/L DZNep (**g**,**h**). Cell colonies were stained with 0.1% crystal violet and the colonies contained were counted manually under a microscope. Error bars represent SD (n = 3). (MTT, Methylthiazolyl tetrazolium).

We further performed MTT assays to explore the influence of EZH2-shRNA on CRC cell proliferation at the time points of 24, 48, and 72 h of transfection. The results revealed that the EZH2‑shRNA group exhibited a markedly reduced proliferation rate compared with the control-shRNA and untreated groups (*p* < 0.05, Figure 4a–d). That is to say, downregulation of EZH2 inhibits cell proliferation in CRC cells. To further examine whether knockdown of EZH2 suppresses migration ability of RKO and HCT116 cells, we performed cell migration assays using transwell cell culture inserts. EZH2-shRNA or DZNep decreased the proliferation of RKO and HCT116 cells. Therefore, to rule out the possibility that the fewer number of viable cells with transmembrane in the EZH2-shRNA group was a result of EZH2’s suppressive effect on cell proliferation, we detected the ability of migration by transwell assay after cells were transfected with EZH2-shRNA or not, or treated with DZNep or not. Images were captured after 24 h (Figure 4e,g). The results from cell migration assay showed that the migration cell number of the EZH2-shRNA transfected group was significantly lower than that of untreated and control-shRNA groups (*p* < 0.05, Figure 4f). The cells treated with DZNep had a similar outcome (*p* < 0.05, Figure 4h). Collectively, these results suggested that the downregulation of EZH2 or addition of DZNep significantly inhibits the migratory capability of RKO and HCT116 cells.

### 3.4. Downregulation of EZH2 Induced Autophagy in RKO and HCT116 Cells

**Figure 5:**
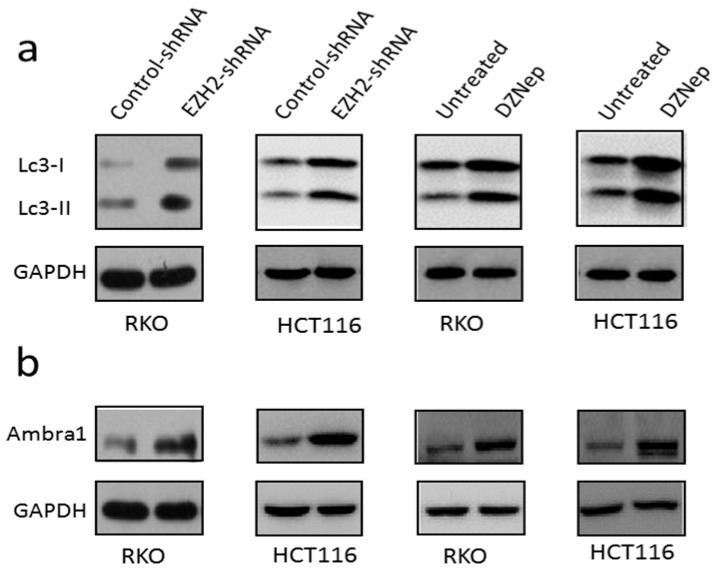
Depletion of EZH2 by specific shRNA induced autophagy in RKO and HCT116 cells. RKO and HCT116 cells were transfected with control-shRNA or EZH2-shRNA or added 3 μmol/L DZNep for 72 h. Western blot for LC3-II (**a**) as well as Ambra1 (**b**) protein levels are shown. GAPDH levels were monitored to ensure equal loading.

To analyze the impact of EZH2 on autophagy, RKO and HCT116 cells were treated with EZH2-shRNA. It is well known that the autophagy is determined by the level of LC3. LC3-I is located in the cytoplasm, when it conjugates to phosphatidylethanolamine, it transforms to LC3-II and recruits to autophagosomal membranes. LC3-II is a widely used marker to monitor autophagy. As presented in Figure 5a,b, we found that compared with control group, knockdown of EZH2 increased the expression of LC3-II significantly. Moreover, the results of adding DZNep showed a similar trend. Moreover, levels of another autophagy-related gene-related protein Ambra1 increased. Taken together, our data suggested that depletion of EZH2 by specific shRNA or relative chemical inhibitor-induced autophagy in RKO and HCT116 cells.

### 3.5. Downregulation of EZH2 Induced Apoptosis in RKO and HCT116 Cells

**Figure 6:**
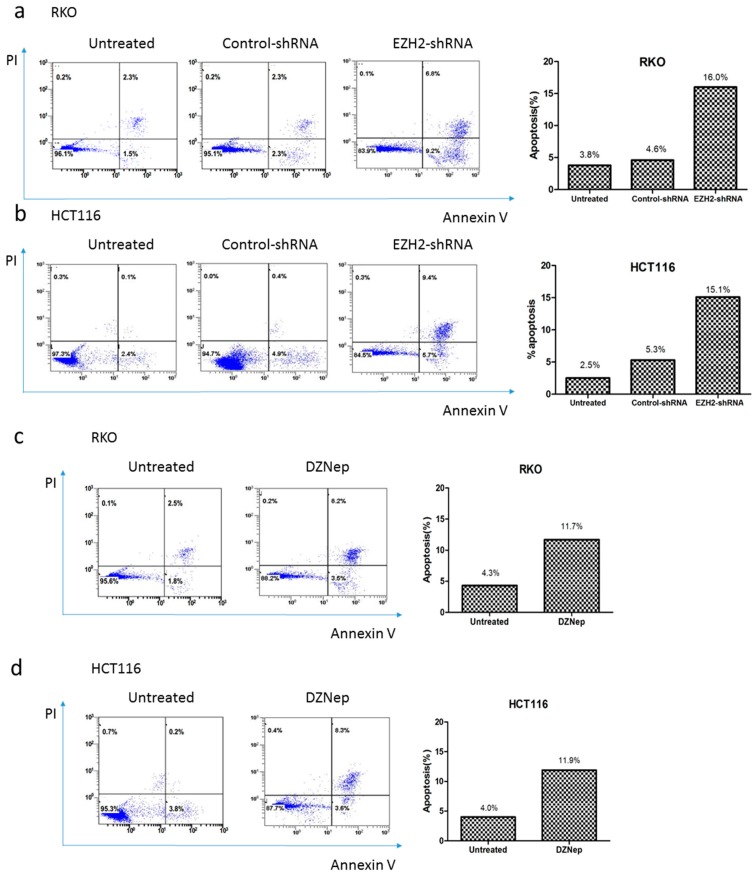
EZH2-shRNA or DZNep alters apoptosis rate in RKO and HCT116 cells. Cells were transfected with EZH2-shRNA or control-shRNA (**a**,**b**), or added 3 μmol/L DZNep (**c**,**d**), 72 h later, cells were stained with Annexin V and PI, and then analyzed by a flow cytometer.

We further performed experiments to evaluate whether depletion of EZH2 by specific shRNA or adding exogenous DZNep affected the apoptosis of RKO and HCT116 cells. As shown in Figure 6, apoptosis rates in EZH2-shRNA group significantly increased when compared with the control-shRNA and untreated groups (*p* < 0.01). After transfection, the apoptosis rate (early and late apoptotic cells) of RKO and HCT116 cells was 16.0% and 15.1% for the EZH2-shRNA group, which was significantly higher than that of the control-shRNA group (4.6% and 5.3%) or untreated group (3.8% and 2.5% respectively). DZNep could exert a similar effect on inducing apoptosis (Figure 6c,d). Thus, these results suggested that depletion of EZH2 by specific shRNA or decrease of the expression of EZH2 by DZNep induced apoptosis in RKO and HCT116 cells.

### 3.6. Downregulation of EZH2 Induced Cell Cycle Arrest in RKO and HCT116 Cells

**Figure 7:**
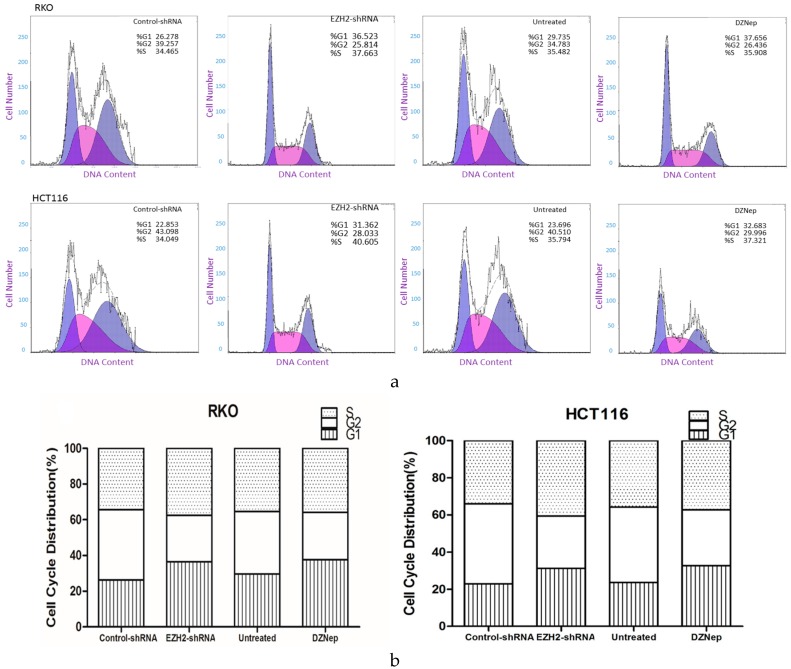
EZH2-shRNA or DZNep altered alters cell cycle distribution in RKO cells. Cells were transfected with EZH2-shRNA or control-shRNA, or added 3 μmol/L DZNep, 5 days later, cells were harvested and then fixed by 75% ice-cold ethanol for 2 h. After rinsing by D-Hanks, cells were stained with PI and analyzed by a flow cytometer. (**b**) is the cartogram of (**a**). The results were analyzed by *t-*test.

To investigate the effect of downregulation of EZH2 on the cell cycle of RKO and HCT116 cells, we used flow cytometry to monitor the percentage of cells that contained DNA at a relative level. As shown in Figure 7, after the treatment with shRNA, the percentage of RKO and HCT116 cells in G1 phase in the EZH2-shRNA group was significantly higher than in the control group, as with DZNep. Meanwhile, the percentage of cells in S and G2 phase is decreased. That is to say, a decrease in the expression level of EZH2 inhibits G1/S transition.

## 4. Discussion

With more and more research available, the epigenetic molecule EZH2 is regarded as a transcriptional repressor associated with many cancer types [14,15,16,17]. Our previous study demonstrated that compared to normal tissues, colorectal cancer tissues expressed higher levels of EZH2. Elevated EZH2 was significantly correlated with TNM stage and lymph node metastasis in CRC tissues [13]. Moreover, many works demonstrate that upregulation of EZH2 is a sign of poor prognosis [10,11,12], which indicate that EZH2 as an oncogene. However, there is disparate point of view that high level expression of EZH2 inhibits aggressive T-acute lymphoblastic leukemia [18], which means EZH2 can also act as a tumor suppressor. In the present study, we investigated the influence of EZH2-shRNA or DZNep on CRC cells. We detected that EZH2 mRNA was highly expressed in CRC cell lines and the proliferation and migration ability of RKO and HCT116 cells was inhibited obviously after downregulation of EZH2. Meanwhile, the autophagy level and apoptotic rate were elevated. These results suggested that EZH2 plays an important role in CRC cell progression.

To evaluate whether EZH2 exerts influence on proliferation via regulation of cell cycle, we performed FACS analysis. The results showed that RKO and HCT116 cells transfected with EZH2-shRNA or treated with DZNep were arrested in G1 phase and decreased the rate of cells in G2 clearly. Thus, EZH2 may play a critical role to regulate the G1/S phase into G2 phase through the cell cycle arrest, which is associated with proliferation during tumor progression and leads to poor prognosis. However, downregulation of EZH2 made cells enter a period of stasis in G1 to inhibit the proliferation of cancer cell lines, which may reverse the condition of tumor and prolong the overall survival of patients [13,19,20,21].

Moreover, the association between EZH2 and apoptosis was observed in RKO and HCT116 cells. Compared to control group, downregulation of EZH2 distinctly increased apoptotic rate of cells. Apoptosis has been recognized as a key system to clear aged or damaged cells, and it is also essential in the process of carcinogenesis. EZH2 plays a specific role in apoptosis and mediates the degree of it in many cancers [22,23,24,25]. Targeting of EZH2, a potential therapeutic strategy, may induce apoptosis and tumor cell death. Furthermore, Zhu Z. et al. [22] considered EZH2 enhance the sensitivity of tumor to cisplatin, which also promotes apoptosis. Wu C. et al. [26] found that inhibition of EZH2 by genetic and pharmacological means sensitizes prostate cancer cells to camptothecin-induced apoptotic death and growth inhibition in culture and in mice. Taken together, EZH2 may repress apoptosis, while downregulation of EZH2 may accelerate apoptosis.

It is evident that autophagy has been recognized as an evolutionarily conserved catabolic process. It is thought to promote cell survival in response to stress by suppressing chronic tissue damage, inflammation, recycling cellular components and maintaining genome stability via its quality control function [27]. Autophagy is reported to play a dual role in cancer cells. On the one hand, autophagy can result in cell death under certain conditions (termed type II programmed cell death) [28]. On the other hand, autophagy sustains tumor metabolism, growth, and survival via nutrient recycling [29]. To date, it remains unclear whether autophagy acts fundamentally as a cell survival or cell death pathway, or both. In our study, we found increased expression of LC3-II and Ambra1 in CRC cell lines after transfection with EZH2-shRNA or addition of exogenous DZNep. LC3-II and Ambra1 are the marker proteins of occurrence of autophagy [30,31,32]. Our previous study evidenced that the increased expression of Ambra1 corresponded to the induction of autophagy [33]. Thus, this mean downregulation of EZH2 may induce autophagy in CRC cells.

More importantly, there are few studies that involve epigenetic gene and autophagy. There are multiple epigenetic mechanisms, such as chromatin modulation, histone modification, and microRNAs (miRNAs) that regulate the autophagy. For example, the activity of histone deacetylase SIRT1 [32] or histone mark H3K4me [34] regulates autophagy. As an epigenetic gene, EZH2 has only been reported to regulate in HCC cells [35] and glioblastoma multiforme [36]. To our knowledge, these findings provided important evidence that epigenetic gene EZH2 regulates autophagy in CRC cells.

The relationship between autophagy and apoptosis, an important question, has been widely studied in recent years. Although apoptosis and autophagy bear different morphological characteristics and physiological processes, they interact each other and sometimes can exert synergetic effects [37]. Apoptosis and autophagy share a lot of same properties. For example, Bcl-2 is a well-known inhibitor of both autophagy and apoptosis [38,39]. Beclin1, VPS34 and ATG5 positively regulate autophagy and negatively regulate apoptosis [40,41,42]. Besides, the same cellular stresses can in some cases activate both autophagy and apoptosis [40]. Taking into account that autophagy and apoptosis are both related to cancerogenesis, our present research provided evidence that downregulation of EZH2 induces both autophagy and apoptosis in CRC cells. To clarify the relationship between autophagy and apoptosis caused by downregulation of EZH2 in CRC cells, further studies should be performed to investigate, for example, the impact of EZH2-shRNA or addition of EZH2 inhibitors on apoptosis rate when autophagy is inhibited.

There are some limitations in our study. Firstly, we didn’t investigate the effect of depletion of EZH2 on colorectal cancer in vivo. Moreover, autophagy is a dynamic process, and we didn′t observe the autophagy flux. And the detailed mechanisms by which apoptosis and autophagy are regulated in CRC cells have not been fully elucidated. However, cell autophagy and apoptosis are complex networks, our research is still not enough to clarify the relationship between EZH2, autophagy and apoptosis. Thus, more research should be undertaken to get a better understanding of epigenetic mechanisms and the influence of EZH2 targeted agents on potential survival benefit in CRC patients, which may promote a novel clinical strategy for CRC treatment. Meanwhile, a variety of inhibitors suppress the EZH2 via different mechanisms, such as regulation of other targets or signal pathways, competing toward the SET domain of EZH2 and inducing relevant protein degradation [43,44,45]. All methods of inhibiting EZH2 seem have a potential benefit for treating solid tumors [45,46,47,48]. For example, GSK126, an EZH2 competitive inhibitor, is undergoing phase I clinical trials for treating hypermethylation-related cancers [49]. However, many studies have also shown that the effect of prior competitive inhibitors is limited, which does not seem to obtain the same effect as knockdown of EZH2. Further research is needed to clarify these mechanisms and to detect the clinical survival benefit in this realm.

In summary, our study has partly unveiled the association of EZH2 with CRC cells. We observed that mRNA and protein of EZH2 were over-expressed in CRC cells. And we also found that the EZH2 level of protein and RNA was remarkably reduced by shRNA. We have further demonstrated that EZH2 has a role in mediating cell proliferation, migration, and restraining cell apoptosis and cell cycle arrest. More importantly, our data showed that EZH2 is related with autophagy. The autophagy induced by downregulation of EZH2 may explain the impact of EZH2 on CRC cell viability. Collectively, our study indicated that downregulation of the epigenetic gene EZH2 may contribute to eliminate the CRC cells, which may provide a potential mechanism for the treatment of colorectal cancer.

## 5. Conclusions

Downregulation of EZH2 or adding exogenous inhibitors of EZH2 is essential for the induction of autophagy and apoptosis in CRC cells. Additionally, decrease of EZH2 also exerts the influence on cell cycle, proliferation and migration of CRC cells. It may provide a potential mechanism for the targeted treatment of colorectal cancer.

## Figures and Tables

**Table 1:** Sequences and target of shRNA template oligonucleotides.

Target sequence 1	TAGGTTAATTGGGACCAAA
Sense strand 5’-3’	CCGGGCTAGGTTAATTGGGACCAAACTCGAGTTTGGTCCCAATTAACC
Antisense strand 5’–3’	AATTCAAAAAGCTAGGTTAATTGGGACCAAACTCGAGTTTGGTCCCAA
Target sequence 2	CAACATAGATGGACCAAAT
Sense strand 5’-3’	CCGGCCCAACATAGATGGACCAAATCTCGAGATTTGGTCCATCTATGTT
Antisense strand 5’–3’	AATTCAAAAACCCAACATAGATGGACCAAATCTCGAGATTTGGTCCAT
Target sequence 3	GAAATCTTAAACCAAGAAT
Sense strand 5’-3’	CCGGCGGAAATCTTAAACCAAGAATCTCGAGATTCTTGGTTTAAG
Antisense strand 5’–3’	AATTCAAAAACGGAAATCTTAAACCAAGAATCTCGAGATTCTTGGTTT
